# *Oswaldocruzia ukrainae* (Nematoda: Molineidae)—A Parasite of European Green Toad *Bufotes viridis*: Morphological and Molecular Data

**DOI:** 10.3390/biology12060772

**Published:** 2023-05-26

**Authors:** Nadezhda Yu. Kirillova, Alexander A. Kirillov, Sergei V. Shchenkov, Igor V. Chikhlyaev

**Affiliations:** 1 Samara Federal Research Scientific Center RAS, Institute of Ecology of Volga River Basin RAS, 445003 Togliatti, Russia; nadinkirillova2011@yandex.ru (N.Y.K.);; 2 Department of Invertebrate Zoology, Saint Petersburg State University, 199034 St. Petersburg, Russia

**Keywords:** amphibia, nematodes, *Oswaldocruzia ukrainae*, trichostrongylids, European green toad, Middle Volga region

## Abstract

**Simple Summary:**

Roundworms of the genus *Oswaldocruzia* are common parasites of the small intestine of amphibians and reptiles. Our recent study revealed that only one species, *Oswaldocruzia filiformis*, parasitizes anurans, lizards and snakes in European Russia. In this work we studied nematodes of the genus *Oswaldocruzia* in the European green toads from the Middle Volga region using traditional morphological and molecular genetic methods. Descriptions, original drawings, and data on molecular phylogenetic analysis for studied nematodes were produced. The results of our study showed that green toads are parasitized by two *Oswaldocruzia* species, *Oswaldocruzia ukrainae* and *O. filiformis*. We found broad variability in morphology of the studied nematodes, which can lead to inaccurate identification of closely related species of *Oswaldocruzia*. The data obtained in our study should contribute to the knowledge of *Oswaldocruzia* nematodes and host-parasite associations in general.

**Abstract:**

Nematodes of the genus *Oswaldocruzia* are common parasites of the small intestine of amphibians and reptiles. Our recent molecular analysis of *Oswaldocruzia* nematodes revealed that only *Oswaldocruzia filiformis*, which possesses high morphological variability, parasitizes amphibians and reptiles in European Russia. Here we present the study of *Oswaldocruzia* nematodes from the European green toad *Bufotes viridis* (Anura, Bufonidae) collected at different localities of the Middle Volga region in 2018–2022. We analyzed the morphological characteristics of the *Oswaldocruzia* spp. taxonomy together with novel molecular phylogenetic data. The data on phylogenetic analysis (based on partial CoxI mtDNA gene sequences) showed that *Bufotes viridis* is parasitized by two *Oswaldocruzia* species, the host-specific parasite *Oswaldocruzia ukrainae* and species generalist *Oswaldocruzia filiformis*. Broad morphological variability was revealed in *O. ukrainae* nematodes both from the same host specimen and from various toad individuals from different localities. Our results highlight the need for further biodiversity research of morphologically similar *Oswaldocruzia* species from amphibians and reptiles in the Western Palearctic using molecular genetic methods.

## 1. Introduction

The distribution range of the European green toad, *Bufotes viridis* Laurenti, 1768 (Amphibia: Bufonidae), runs from eastern France to the Balkans and European Russia. Previously, this toad species also inhabited the Middle East and Central Asia, reaching western China and northwestern India, as well as in the south to North Africa. Nowadays, toad populations inhabiting North Africa, the Balearic Islands and Asia are considered separate species [1,2].

Nematodes from the genus *Oswaldocruzia* Travassos, 1917 are one of the most common parasites of *B. viridis*. These nematodes parasitize the small intestine of amphibians and reptiles, mainly in anurans. This genus includes about 90 species distributed worldwide [3,4,5,6,7,8,9,10,11,12,13,14,15,16,17,18,19,20,21,22,23,24,25,26,27,28,29,30,31,32,33,34,35,36,37].

In Russia, three species, *Oswaldocruzia filiformis* (Goeze, 1782), *Oswaldocruzia ukrainae* Iwanitzky, 1928, and *Oswaldocruzia iwanitzkyi* Sudarikov, 1951, were found in *B. viridis* [3,38,39]. Nematodes of the genus *Oswaldocruzia* have broad morphological variability [40,41,42], which may lead to inaccurate species affiliation of *Oswaldocruzia* spp. from various host species. As a result, a number of previously described *Oswaldocruzia* species were proposed to be reduced as synonyms of the first described species, *O. filiformis* [3,33,43].

In most cases, only *O. filiformis* was recorded in the green toad *B. viridis* from various regions of European Russia. This nematode species was identified in *B. viridis* from the Republic of Tatarstan [44,45], Republic of Bashkortostan [46,47,48,49], Tambov [50], Samara [51,52,53,54,55], Nizhny Novgorod [56], Orenburg [57,58] and Astrakhan [38,39] regions. Outside of Russia, *O. filiformis* has been found in *B. viridis* from Ukraine [3], Belarus [59,60], Bulgaria [61], Turkey [14,25,26,62] and Uzbekistan [13,63].

Ivanitsky [64] described three species of *Oswaldocruzia* in *B. viridis*, namely *O. fulleborni*, *O. ukrainae* and *O. skrjabini*. Sudarikov [65] revised the latter species as *Oswaldocruzia iwanitzkyi* Sudarikov, 1951. According to Ryzhikov et al. [3], *O. fulleborni* is morphologically very similar to *O. filiformis*.

In Russia, the nematode *O. ukrainae* was previously identified in *B. viridis* only in the Stavropol, Volgograd and Astrakhan regions [3,38,39]. *Oswaldocruzia ukrainae* has also been recorded in toads from the Poltava region of Ukraine [33], Uzbekistan [13,63] and Tunisia [4]. In addition, *O. iwanitzkyi*, a new species for Russia, was revealed in *B. viridis* from the Astrakhan region [38,39]. This species has previously been recorded only in green toads of Ukraine [64] and Slovakia [66]. During the helminthological study of toads in the type area, Svitin [33] found only *O. ukrainae* in *B. viridis*. Therefore, the validity of *O. iwanitzkyi* requires clarification.

The species *O. ukrainae* is a parasite specific to *B. viridis* and has not been recorded in other amphibian species. On the contrary, the nematode *O. filiformis* is a species generalist parasitizing most species of amphibians in the Palaearctic. This species greatly exceeds all other nematodes of amphibians in terms of the number of hosts. Twelve amphibian species have been registered as hosts for *O. filiformis* in Russia and neighboring countries [3,52]. 

We have previously identified *Oswaldocruzia* nematodes from various amphibian species in the Middle Volga region as *O. filiformis* [35,55,67,68,69]. Our recent molecular and morphological analysis of *Oswaldocruzia* nematodes revealed that only one species, *O. filiformis*, parasitizes amphibians and reptiles in European Russia [40]. Unfortunately, that study did not include *Oswaldocruzia* specimens parasitizing the European green toads, *B. viridis*.

The purpose of this study was species identification of *Oswaldocruzia* nematodes parasitizing *B. viridis* in European Russia using morphological and molecular phylogenetic analysis. We also compared the newly obtained results with our previous data.

## 2. Materials and Methods

### 2.1. Nematode Collection and Examination

**Figure 1:**
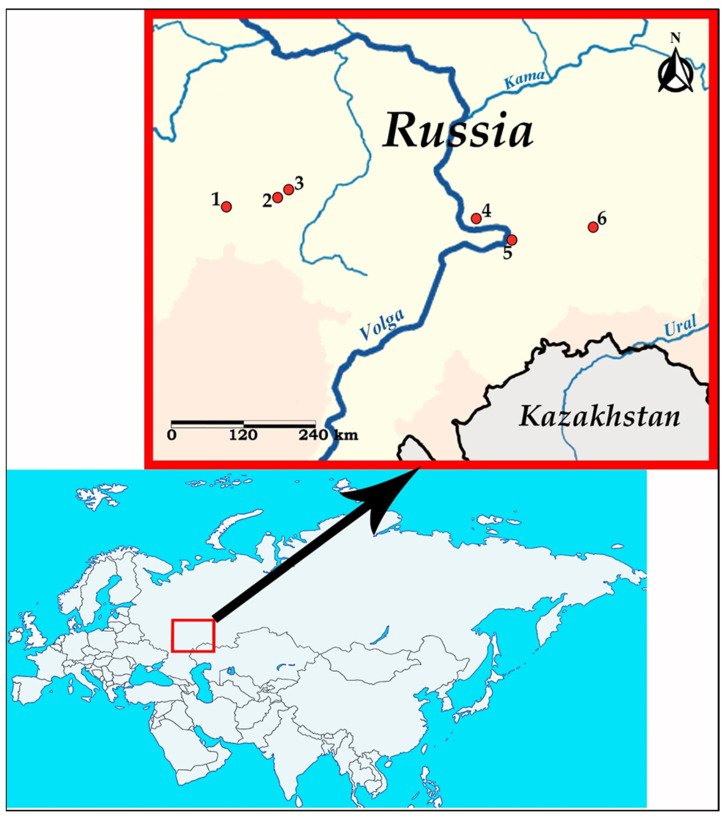
Map of sampling sites in the Middle Volga region (European Russia). Red circles indicate sampling localities: 1—Vicinity of Spassk (Penza region); 2—Nikolaevka village (Republic of Mordovia); 3—Lykovshina village (Republic of Mordovia); 4—Togliatti (Samara region); 5—Samara city; and 6—Oktyabrskiy township (Samara region).

Nematodes of the genus *Oswaldocruzia* were collected from the small intestine of *B. viridis* at six localities in the Middle Volga region (European Russia) in 2018–2022: vicinity of Spassk city (Penza region, 53°54′49″ N, 43°12′36″ E), Nikolaevka village (Republic of Mordovia, 54°8′34″ N, 45°8′55″ E), Lykovshina village (Republic of Mordovia, 54°18′45″ N, 45°29′13″ E), Togliatti city (Samara region, 53°33′6″ N, 49°12′40″ E), Samara city (53°12′27″ N, 50°9′18″ E) and Oktyabrskiy township (Samara region, 53°25′33″ N, 52°3′48″ E). Data on the geographic origin of the examined *Oswaldocruzia* nematodes are presented in Figure 1 and Table 1.

No amphibians were intentionally killed for this research. In this work, we studied only road-killed toads. Several dead amphibians were kindly provided by the rural residents. The necropsy was performed on toads approximately 1–8 h after their death. Only alive motile adult nematodes were collected for further investigation. Some nematodes were collected from toads and preserved in 96% ethanol for further molecular phylogenetic analysis. For the morphological analysis, the nematodes were killed by heating in water, then parasites were cleared in lactic acid. In this work we studied and measured 59 *Oswaldocruzia* nematodes, including 26 males and 33 females.

Microscopic examination, morphological measurements and drawings of the nematodes were performed using MBI-9 light microscope, drawing tube RA-7 and the Levenhuk M500 BASE Digital Camera. We made apical and transverse nematode sections manually using a razor blade.

The synlophe and spicules were described in accordance with Durette-Desset [72]. The nomenclature of the male caudal bursa is specified following Durette-Desset and Chabaud [73]. The studied morphological characteristics were the shape of cephalic vesicle, the shape of lateral alae at mid-esophagus level, the number of crests in the level of mid-body, the shape and structure of male caudal bursa, the shape of male spicules, the shape and structure of the dorsal ray of bursa. We also examined such morphometric features as body length, width at mid-body level, length of esophagus, length and width of cephalic vesicle, tail length, spicule length, egg length and width, and distances from the anterior end of the body to the vulva, excretory pore and nerve ring.

### 2.2. DNA Extraction, Amplification, Sequencing, and Phylogenetic Analysis

In order to obtain partial CoxI mtDNA sequences, seven specimens of ethanol-fixed *Oswaldocruzia* were dried at 37 °C in a dry block heater for 1 h. The specimens were then transferred to clear 500 µL tubes with a mixture of 48 µL 0.1% Chelex-100 and 2 µL Proteinase K (concentration 10 mg/mL) and incubated for 2 h at 55 °C and 25 min at 95 °C to stop proteinase activity. After that, the water solution of the total DNA was transferred to a sterile 500 µL tube and frozen at −20 °C. The amplification primer pair was JB3 (5′-TTT TTT GGG CAT CCT GAG GTT TAT-3′) and JB4,5 (5′-TAA AGA AAG AAC ATA ATG AAA ATG-3′) [74]. PCR was performed in 25 µL of reaction mix per PCR-tube in a BioRad C1000 thermal cycler. The following parameters were used: initial denaturation (3 min at 95 °C) followed by 35 cycles of 20 s at 95 °C, 20 s at 53 °C and 40 s at 72 °C, with 5 min at 72 °C for final extension [40]. Amplicons were directly sequenced using ABI-PRISM 3500xl sequencer (Life Technologies, Carlsbad, Massachusetts, USA) with the same primers. The sequences were mounted in general alignment with other nematode species, which were downloaded from GenBank NCBI using a custom R script [75,76] (Table 1). The sequences were automatically aligned using Muscle algorithm [77] as implemented in SeaView 4.0 [78]; the alignment was then trimmed manually. The phylogenetic analysis was performed using the maximum likelihood method at Cipres portal [79] with GTR+G+I model and a non-parametric bootstrap with 1000 pseudoreplicates. Bayesian analysis was performed with MrBayes on XSEDE 3.2.7a (GTR+G+I model). The model for phylogenetic analysis was chosen with MrModeltest 2.3 [80]. Trees were run as two separate chains (default heating parameters) for 15,000,000 generations. The quality of the chains was estimated using built-in MrBayes tools and, additionally, using the Tracer 1.6 package [81]. Based on the estimates, the first 25,000 generations were discarded for burn-in.

The *p*-distances were calculated using MEGA11 software [82] with standard parameters. Results of *p*-distance estimation were used as input data for ‘ComplexHeatmap’ library in R programming language [76,83].

## 3. Results

We found the nematodes *O. ukrainae* during a helminthological study of *B. viridis* from various localities of the Middle Volga region (Figure 1). These specimens were identified in toads from the Republic Mordovia and Samara region. At the same time, during the study of one individual of *B. viridis* from the vicinity of the Spassk city (Penza region), one male of *O. filiformis* was revealed.

### 3.1. Morphological Description of Nematodes

#### 3.1.1. Morphology of *Oswaldocruzia ukrainae*

**Figure 2:**
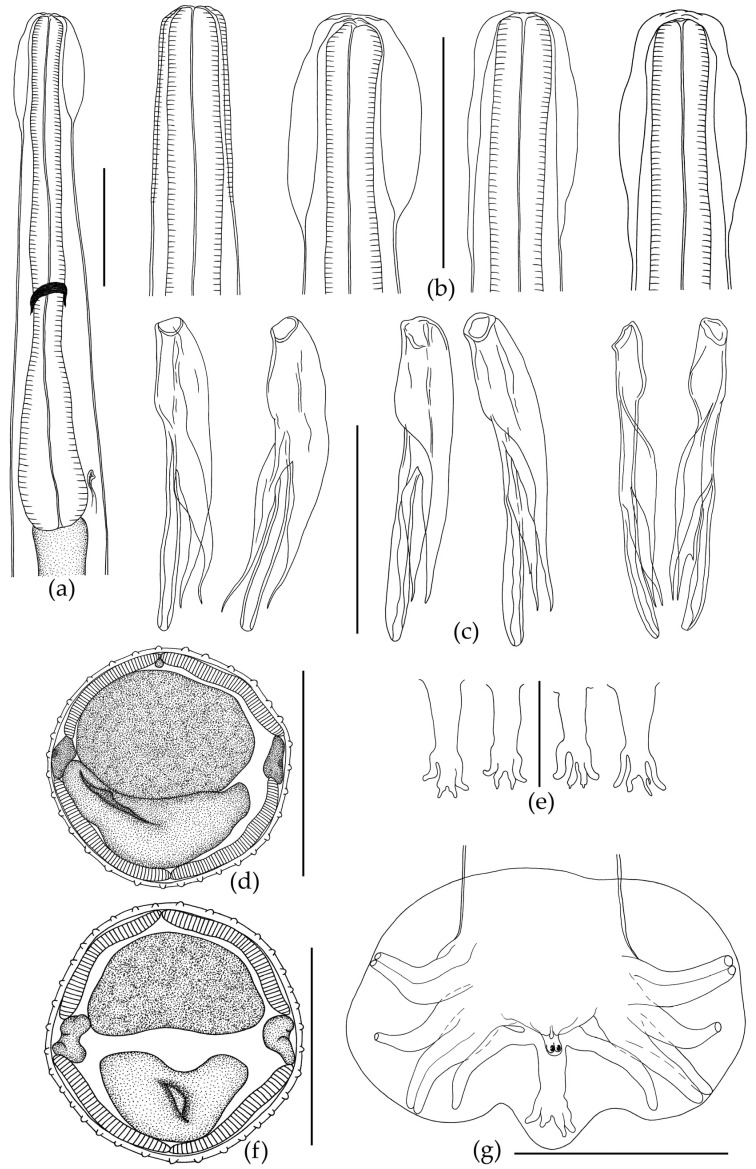
*Oswaldocruzia ukrainae* male from *Bufotes viridis*. (**a**) Anterior body end, lateral view; (**b**) variation of cephalic vesicle; (**c**) spicules from different male specimens; (**d**) transverse section at mid-body, 32 crests; (**e**) variability of dorsal ray of bursa; (**f**) transverse section at mid-body, 35 crests; (**g**) caudal bursa, ventral view. (**a**–**d**,**f**,**g**) Scale bar, 0.1 mm; (**e**) scale bar, 0.05 mm.

**Figure 3:**
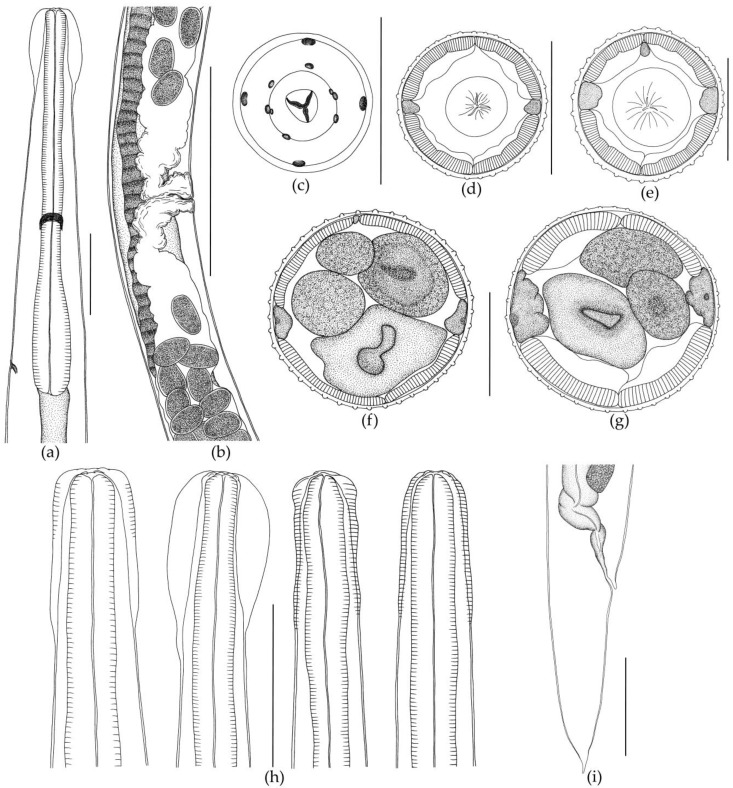
*Oswaldocruzia ukrainae* female from *Bufotes viridis*. (**a**) Anterior body end, lateral view; (**b**) region of vulva and ovejector, left lateral view; (**c**) head, apical view; (**d**) transverse section at the level of mid-esophagus, 33 crests; (**e**) transverse section at the level of mid-esophagus, 35 crests; (**f**) transverse section at the level of mid-body, 42 crests; (**g**) transverse section at the level of mid-body, 46 crests; (**h**) variability of cephalic vesicle; (**i**) tail, left lateral view. (**a**,**f**–**i**) Scale bar, 0.1 mm; (**b**) scale bar, 0.5 mm; (**c**,**f**,**e**) scale bar, 0.05 mm.

The body of the nematode is thin and elongated, with maximum width at mid-length. The anterior body end has a cephalic vesicle that is variable in shape (Figure 2b and Figure 3h).

Shape of vesicle is variable even in specimens of *O. ukrainae* from one individual toad. The cuticle forms continuous longitudinal ridges beginning behind the cephalic vesicle and running along the whole nematode body, forming a synlophe, which is symmetrical. Lateral alae are absent (Figure 3). The number of crests at mid-length of the body varies depending on sex of nematodes (Figure 2 and Figure 3, Table 2 and Table 3). Ridges are not continuous from the anterior end of body to the tail. They can end in one area, and then appear in another body part. The anterior end of the body is rounded. The triangular oral opening is surrounded by four large cephalic papillae and six external-labial papillae, of which the lateral papillae are close to small, not always distinguishable amphids (Figure 3). The esophagus is club-shaped, thin, cylindrical in the anterior part and wide in the posterior part. The posterior end of the esophagus is rounded with a posterior bulb (Figure 2 and Figure 3). Excretory pore position varies within the posterior third of the esophagus. The nerve ring surrounding the esophagus in the middle part is slightly closer to its posterior third (Figure 2 and Figure 3).

Male. The body ends with a wide caudal bursa that is symmetrical and three-lobed (two lateral and one dorsal). According to the classification of Durette-Desset and Chabaud [73], it belongs to type II. Ray 9 in male bursa always have a more or less S-shaped bend. Ray 10 always has extra branches of variable size (Figure 2). The genital cone is well developed, with two papillae (Figure 2). Gubernaculum is absent. Spicules are approximately equal and surrounded by a thin membrane. According to Durette-Desset [72], spicules are non-idiomorphic, with three branches: the internal branch is wide in the anterior part and narrows towards the posterior sharp end (Figure 2). The central branch is the longest, is rounded at the end, and has no extra offshoots. The external branch is narrow, sharp at the end; at the level of the last third it has a small offshoot (Figure 2). Measurements are presented in Table 2.

Female. The body is always larger than in males, tapering to the posterior end. The tail has a thin tip (Figure 3). The female reproductive system, like in all *Oswaldocruzia* species, is amphidelphic. Vulva is wide, opening postequatorially, behind the body at mid-length. The muscular vagina passes into paired ovejectors, which have powerful sphincters (Figure 3). The anterior ovary forms numerous loops in the anterior body part. The ovary reaches the level of esophagus and returns. The posterior ovary extends to the body end, strongly bends and turns forward slightly twisting, going beyond the level of the vulva. The uterus of adult females is filled with eggs. All eggs in vulva, ovejector and uterus were found at morula stage. Measurements of the main characteristics are presented in Table 3.

#### 3.1.2. Morphology of *Oswaldocruzia filiformis* Male

**Figure 4:**
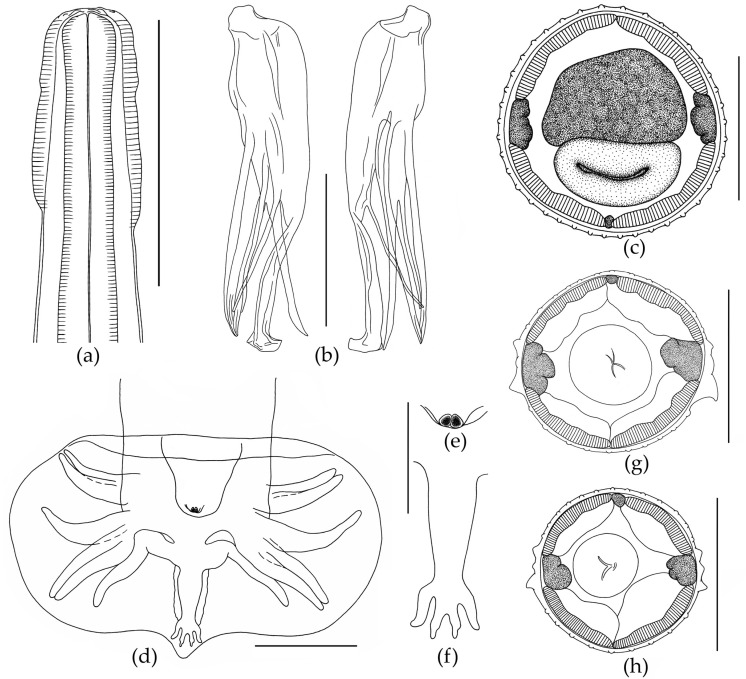
*Oswaldocruzia filiformis* male from *Bufotes viridis*. (**a**) Cephalic vesicle; (**b**) spicules, lateral view; (**c**) transverse section at the level of mid-body, 40 crests; (**d**) caudal bursa, ventral view; (**e**) genital cone, ventral view; (**f**) dorsal ray of bursa; (**g**,**h**) transverse section at the level of mid-esophagus. (**a**–**f**) Scale bar, 0.1 mm; (**g**,**h**) scale bar, 0.05 mm.

The body is thin and elongated. The cephalic vesicle presents on the anterior body end (Figure 4). The cuticle forms longitudinal ridges starting behind the cephalic vesicle and running along the whole body. The anterior end of the body is rounded. The triangular oral opening has four large cephalic papillae and six externo-labial papillae. The esophagus is thin, cylindrical in the anterior part and wide in the posterior part. The posterior end of the esophagus forms a bulb (Figure 4).

The excretory pore is located at the level of the posterior third of the esophagus. The esophagus is surrounded by a nerve ring at the level of its middle part. Synlophe is symmetrical. On transverse sections, at the level of the second third of the esophagus, small cervical alae are clearly visible, formed by three slightly enlarged crests (Figure 4). Lateral alae consist of three ridges, with a more developed ventral crest. Two much smaller ridges (dorsal and central) are directly above the large ventral crest (Figure 4). At the level of the anterior part of the intestine, the lateral alae transform into simple ridges. The number of crests at mid-length of the body is 40 (Figure 4). A wide caudal bursa is at the end of the body. The caudal bursa is three-lobed, symmetrical and belongs to type II [73]. Ray 9 is in the form of an S-shaped bend. Extra branches on ray 10 are not expressed (Figure 4). The genital cone is well developed and has two papillae (Figure 4). According to Durette-Desset [72], spicules are idiomorphic, approximately equal and surrounded by a thin membrane. Each spicule has three branches, namely “blade”, “fork” and “shoe”. The end of the blade is visually divided into two branches, as well as distal parts of branches that visually split into two narrow offshoots. In fact, the branches do not split, but are tightly pressed against each other. The fork is divided into two branches approximately at mid-length. The shoe has a thin branch at mid-length and is slightly bent at its distal end (Figure 4). Measurements are presented in Table 4.

### 3.2. Molecular Phylogenetic Analysis

**Figure 5:**
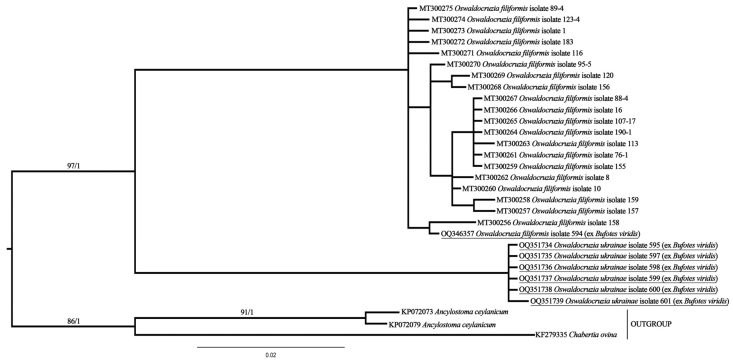
Data on the molecular phylogenetic analysis of *Oswaldocruzia* spp. based on CoxI mtDNA gene sequences. ML/BI values are shown. Supports of deeply branched clades are not given due to its polyphyly and several mismatches between ML and BI analysis.

According to the results of molecular phylogenetic analysis, two clusters of specimens with almost full support are clearly distinguishable (Figure 5).

One cluster includes specimens of *O. filiformis*, and the other is a clade of *O. ukrainae*. One of the newly obtained sequences, belonging to *O. filiformis*, clustered together with all previously generated ones, although this specimen was collected from *B. viridis*, a typical host for *O. ukrainae*.

**Figure 6:**
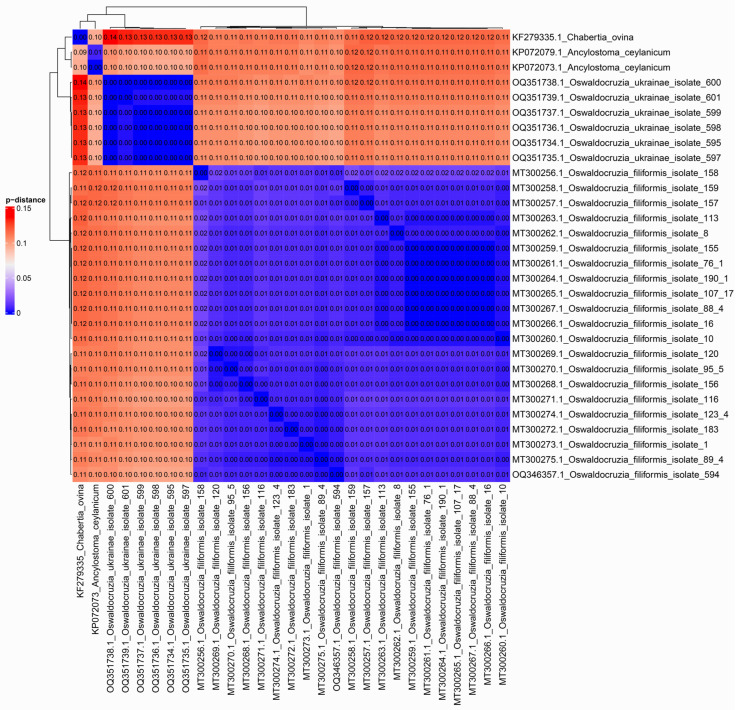
Heatmap based on pairwise distances between CoxI mtDNA sequences of specimens under consideration. Numeric values of *p*-distances were translated into color: the greater the differences, the warmer the color. The specific values in the comparison pairs are indicated in the cells.

The presence of two clusters is also confirmed by the results of *p*-distance estimation. On average, the *p*-distance among specimens of the same species is about 0.01, but approximately 0.11 between *O. filiformis* and *O. ukrainae* (Figure 6). As in phylogenetic reconstruction, a single specimen of *O. filiformis* collected from *B. viridis* is clustered together with other representatives of this species from different hosts.

## 4. Discussion

Here we present a morphological description of two species of *Oswaldocruzia* nematodes of the European green toad *Bufotes viridis* inhabiting the Middle Volga region (European Russia) and new molecular phylogenetic data for these species. The combined use of both molecular and morphological methods allowed the reliable identification of *Oswaldocruzia* spp.

Morphological and molecular data obtained in our work showed that *Bufotes viridis* in European Russia is parasitized by two species of the *Oswaldocruzia* genus, *O. ukrainae* and *O. filiformis*. *Oswaldocruzia ukrainae* is the only species of the genus in the Western Palearctic with non-idiomorphic spicules formed by three branches [33,72]. The nematode specimens examined in our work correspond to earlier morphological descriptions of *O. ukrainae* [3,33,64].

Some differences in the morphometric characteristics of the nematodes *O. ukrainae* were revealed, as in previous studies of *O. filiformis* [40,41,42]. *Oswaldocruzia ukrainae* specimens from different *B. viridis* individuals collected at various localities of the Middle Volga region and even from one host individual also exhibit broad morphometric variability. We found variability of such morphometric characteristics as the body size, cephalic vesicles and spicules, the distances from the anterior end of the body to the excretory pore, nerve ring and vulva (Table 2 and Table 3). Variability in the number of ridges in the nematode mid-body was also revealed. In this study, the number of ridges in *O. ukrainae* males varies from 32 to 37, as well as in females from 40 to 48 (Table 2 and Table 3). We determined that not all crests extend continuously from the anterior body end to the tail. Some ridges may disappear and not reach the posterior body end. At the same time, new crests may appear elsewhere in the body. This may be one of the reasons for previously noticed variations in the number of crests. Moreover, the number of crests in *Oswaldocruzia* nematodes varies in young and mature individuals as well as in males and females, as we have shown earlier [40,41,42]. We believe that this is directly correlated with the size of *Oswaldocruzia* nematodes. Therefore, the largest *Oswaldocruzia* females will always have the maximum number of ridges in the mid-body. This is also revealed in the case of the nematode age. Thus, young small-sized nematodes will always have fewer crests than large adults. Egg sizes remained relatively stable (Table 2 and Table 3). Variability in the morphology of different *O. ukrainae* individuals was also revealed, as in the case of *O. filiformis* [40,41,42]. Thus, the shape of the head vesicle in individuals of both sexes varies greatly in nematodes from different toad individuals and from the same host specimen (Figure 2).

The spicules in *O. ukrainae* males were similar in structure and shape, but we noted differences in their sizes from different nematode individuals (Table 2). The shape and structure of the caudal bursa in *O. ukrainae* males were constant. However, the shape and structure of the dorsal ray of the bursa, which includes the 9 and 10 rays, are varied. All *O. ukrainae* males had an extra offshoot on the 10th ray of different size and shape (Figure 2). In females, the distance from the anterior end of the body to the vulva varied broadly and depended on the total length of the nematodes. Expectedly, this index was the highest in the largest nematode females (Table 3). In addition to *O. ukrainae* specimens, we also revealed one *O. filiformis* male in the examined individuals of *B. viridis* (Table 2 and Figure 3). Previously, we recorded *O. filiformis* in *B. viridis* from European Russia [42]. The description of specimen of *O. filiformis* studied in our work did not differ from the previous morphological descriptions of this species [3,33,40,41,42]. Thus, we confirmed the possibility of parasitism of both *Oswaldocruzia* species in the green toad *B. viridis*. Previously, the parasitism of these nematode species in *B. viridis* was reported by Ivanitsky [64], Ryzhikov et al. [3], Vashetko and Siddikov [13], Andreev [38] and Ikromov [63].

Thus, findings of *O. filiformis* in *B. viridis* are not uncommon and are probably related to the results of syntopic habitation with other amphibian species, especially during the breeding season in semiaquatic habitats. Infection of *B. viridis* with the nematode *O. filiformis* can also occur in habitats where it lives together with the Common toad, *Bufo bufo* (Linnaeus, 1758). 

*Bufotes viridis* and *B. bufo* are sympatric species with overlapping ranges. In European Russia, the territory of the joint habitat of both toad species coincides with the boundaries of the forest-steppe zone. Both toad species lead the same ground-burrow, twilight lifestyle. These synanthropic species inhabit the same anthropogenic environments such as parks, gardens, cellars and agricultural landscapes, and often prefer them to natural habitats [52,84,85,86,87,88]. The similarity in lifestyles contributes to the infection of toads with common helminth species, including the nematode *O. filiformis*. Nematodes of this genus have a direct life cycle. Therefore, simple oral contact of toads with infective nematode larvae on a moist substrate, for example, feeding on terrestrial invertebrates, is sufficient to infect the host.

Ivanitsky [64] described another species of *Oswaldocruzia* that parasitizes *B. viridis* on the territory of Ukraine, *O. fulleborni*, which has not been recorded since the initial description. The main differences of the species were the following: the absence of lateral alae; synlophe consists of 30–50 crests; the “blade” branch is rounded and does not split at the end. We have previously noted varying degrees of the lateral alae development in *O. filiformis* from various hosts and even from the same host specimen [40,41,42]. It should be noted that the lateral alae are clearly visible only on transverse sections of nematodes, which are not presented in Ivanitsky’s work [64]. We also showed that the number of ridges in *O. filiformis* individuals can vary depending on the sex and age of the nematodes themselves. Thus, the number of ridges in mid-body of males of *O. filiformis* from different amphibian species can vary from 33 to 58; and in that of females—from 39 to 78 [40,41,42]. Therefore, these two morphological characteristics cannot be used in the diagnosis of *Oswaldocruzia* species. According to the description and drawing of the spicules of *Oswaldocruzia* males by Ivanitsky [64], we did not find any differences compared to *O. filiformis*. The visible shape of the “blade” end of the branch varies depending on the position of the spicule. We already noted that the distal parts of the “blade” branch are divided into two thin offshoots, but it is not always clearly visible [40]. In fact, these branches are always tightly adjacent to each other. We did not observed separation or splitting of these branches on any of the spicules of *O. filiformis* males [40,41,42].

Judging by the description of *O. fulleborni* by Ivanitsky [64], Ryzhikov et al. [3] believed that there are no significant differences between *O. filiformis* and *O. fulleborni*, and considered these species to be synonymous. Ben Sliman with coauthors [7] considers this species valid due to the difference in the crest number at the level of the mid-body and the structure of the “blade” branch of the spicule. Nevertheless, we agree with the opinion of Ryzhikov et al. [3] and consider *O. fulleborni* to be a synonym of *O. filiformis*, until the opposite is proven. It should also be noted that Svitin [33] found only *O. ukrainae* in the green toads during the study of *Oswaldocruzia* from the type area of *O. fulleborni* and other parts of *Bufotes viridis* range. Due to morphological similarity with *O. filiformis*, the species *O. ukrainae* may be misidentified. Therefore, diagnosis of both species should take into account the morphological and morphometric variability. Apparently, the species *O. ukrainae* could be misdiagnosed as *O. filiformis* in most of the early studies of *Oswaldocruzia* nematodes from *B. viridis*.

When identifying *Oswaldocruzia* nematodes, special care should be paid to the presence/absence of lateral alae. This cuticular structure can be deformed during collection, fixation, or production of whole mounts. Therefore, it is necessary to make transverse sections of nematodes. As our studies have shown, the number of crests also cannot be a diagnostic feature, since the ranges of the number of crests in the two nematode species overlap (Table 2, Table 3 and Table 4) [40,42].

There are also relative differences in body size between the two nematode species. Adult males and females of *O. ukrainae* are somewhat smaller than mature *O. filiformis* individuals. Thus, adult *O. ukrainae* males were not found larger than 7.83, and the body length of the smallest males was 5.00 (Table 2). The size of adult *O. filiformis* males from different species of amphibians was 6.25–13.25 [40,42]. The females of *O. ukrainae* in our study were 9.25–11.55 in length (Table 3), while the body sizes of adult *O. filiformis* females were 9.96–24.50 [40,42]. Thus, the body sizes of the nematodes should be taken into account, but not as a main feature, since they overlap. The only reliable morphological features in the diagnosis of *O. ukrainae* and *O. filiformis* are the size and structure of the male spicules (Figure 2 and Figure 3, Table 3 and Table 4) [40,42].

## 5. Conclusions

Our morphological and molecular phylogenetic data showed that the European green toad, *Bufotes viridis*, in the Middle Volga region is parasitized by two species of the genus *Oswaldocruzia*, the host-specific parasite *O. ukrainae* and species generalist *O. filiformis*. We assessed morphological characteristics for a reliable identification of two closely related *Oswaldocruzia* species under consideration and showed that only size and structure of male spicules can serve as the species diagnosis. The analysis of the morphological features of *O. ukrainae* from *B. viridis* both from various toad individuals and from the same host specimen reveals broad morphological and morphometric variability. In addition, high variability in *O. ukrainae* nematodes was revealed in the green toads from different geographical localities. The obtained data expand our knowledge of amphibian nematodes. We presented the first record of *O. ukrainae* parasitizing amphibians of the Middle Volga region. The results of our studies emphasize the need to confirm the validity of *Oswaldocruzia* species by molecular methods.

## Figures and Tables

**Table 1:** List of *Oswaldocruzia* specimens, with museum and laboratory specimen numbers and GenBank accession numbers according to the geographic origin.

Nematode Species, Isolate No.	Locality	Coordinates	Host	GenBank Acc. No.	Museum and Laboratory Specimen No.	Source
*Oswaldocruzia ukrainae*, 599, 600	Nikolaevka village, suburb of Saransk, Republic of Mordovia	54°8′34″ N, 45°8′55″ E	*Bufotes viridis*	OQ351737 OQ351738	Nem-Os333-599, Nem-Os-334-600	This study
*Oswaldocruzia ukrainae*, 597, 598	Lykovshchina village, Republic of Mordovia	54°18′45″ N, 45°29′13″ E	*Bufotes viridis*	OQ351735 OQ351736	Nem-Os-331-597, Nem-Os-332-598	This study
*Oswaldocruzia ukrainae*, 595, 601	Samara city	53°12′27″ N, 50°9′18″ E	*Bufotes viridis*	OQ351734 OQ351739	Nem-Os-329-595, Nem-Os-335-601	This study
*Oswaldocruzia filiformis*, 594	Vicinity of Spassk city, Penza region	53°54′49″ N, 43°12′36″ E	*Bufotes viridis*	OQ346357	Nem-Os-328-594	This study
*Oswaldocruzia filiformis*, 116	Samarskaya Luka National Park, Samara region	53°10′34″ N, 49°26′12″ E	*Pelophylax ridibundus*	MT300271	Nem-Os-116	[40]
*Oswaldocruzia filiformis*, 120	Samarskaya Luka National Park, Samara region	53°10′34″ N, 49°26′12″ E	*Pelophylax ridibundus*	MT300269	Nem-Os-120	[40]
*Oswaldocruzia filiformis*, 183	Samarskaya Luka National Park, Samara region	53°10′34″ N, 49°26′12″ E	*Pelophylax ridibundus*	MT300272	Nem-Os-183	[40]
*Oswaldocruzia filiformis*, 155	Smolny National Park, Republic of Mordovia	55°50′07″ N, 45°22′43″ E	*Bufo bufo*	MT300259	Nem-Os-155	[40]
*Oswaldocruzia filiformis*, 156	Smolny National Park, Republic of Mordovia	55°45′36″ N, 45°24′21″ E	*Rana arvalis*	MT300268	Nem-Os-156	[40]
*Oswaldocruzia filiformis*, 159	Zvenigorod Biol. Station of Moscow University, Moscow region	55°42′02″ N, 36°43′20″ E	*Rana temporaria*	MT300258	Nem-Os-159	[40]
*Oswaldocruzia filiformis*, 157	Uzola floodplain, Nizhny Novgorod region	56°36′12″ N, 43°35′06″ E	*Rana arvalis*	MT300257	Nem-Os-157	[40]
*Oswaldocruzia filiformis*, 158	Ural floodplain, Orenburg region	51°13′05″ N, 58°33′22″ E	*Rana arvalis*	MT300256	Nem-Os-158	[40]
*Oswaldocruzia filiformis*, 16	Mordovia Nature Reserve, Republic of Mordovia	54°42′49″ N, 43°13′39″ E	*Lacerta agilis*	MT300266	Nem-Os-16	[40]
*Oswaldocruzia filiformis*, 8	Mordovia Nature Reserve, Republic of Mordovia	54°42′49″ N, 43°13′39″ E	*Lacerta agilis*	MT300262	Nem-Os-18	[40]
*Oswaldocruzia filiformis*, 10	Mordovia Nature Reserve, Republic of Mordovia	54°42′49″ N, 43°13′39″ E	*Zootoca vivipara*	MT300260	Nem-Os-10	[40]
*Oswaldocruzia filiformis*, 16_1	Mordovia Nature Reserve, Republic of Mordovia	54°42′49″ N, 43°13′39″ E	*Zootoca vivipara*	MT300273	Nem-Os-16_1	[40]
*Oswaldocruzia filiformis*, 88_4	Smolny National Park, Republic of Mordovia	54°44′49″ N, 45°15′46″ E	*Zootoca vivipara*	MT300267	Nem-Os-88_4	[40]
*Oswaldocruzia filiformis*, 107_17	Smolny National Park, Republic of Mordovia	54°43′58″ N, 45°16′16″ E	*Lacerta agilis*	MT300265	Nem-Os-107_17	[40]
*Oswaldocruzia filiformis*, 95_5	Smolny National Park, Republic of Mordovia	54°43′58″ N, 45°16′16″ E	*Lacerta agilis*	MT300270	Nem-Os-95_5	[40]
*Oswaldocruzia filiformis*, 89_4	Smolny National Park, Republic of Mordovia	54°44′49″ N, 45°15′46″ E	*Lacerta agilis*	MT300275	Nem-Os-89_4	[40]
*Oswaldocruzia filiformis*, 190_1	Smolny National Park, Republic of Mordovia	54°44′41″ N, 45°30′08″ E	*Anguis fragilis*	MT300264	Nem-Os-190_1	[40]
*Oswaldocruzia filiformis*, 76_1	Smolny National Park, Republic of Mordovia	54°44′41″ N, 45°30′08″ E	*Natrix natrix*	MT300261	Nem-Os-76_1	[40]
*Oswaldocruzia filiformis*, 113	Smolny National Park, Republic of Mordovia	54°44′49″ N, 45°15′46″ E	*Vipera berus*	MT300263	Nem-Os-113	[40]
*Oswaldocruzia filiformis*, 123_4	Smolny National Park, Republic of Mordovia	54°44′49″ N, 45°15′46″ E	*Vipera berus*	MT300274	Nem-Os-123_4	[40]
*Chabertia ovina*	Jingyang County, China ^1^	34°31′45″ N, 108°50′24″ E	*Capra aegagrus hircus*	KF279335	–	[70]
*Ancylostoma ceylanicum*	Guangzhou, Guangdong, China ^1^	23°07′49″ N, 113°15′33″ E	*Felis catus* (faeces)	KP072073	–	[71]
*Ancylostoma ceylanicum*	Guangzhou, Guangdong, China ^1^	23°07′49″ N, 113°15′33″ E	*Felis catus* (faeces)	KP072079	–	[71]

^1^ Mean geographical coordinates; authors of this study collected and sequenced the specimens with accession numbers OQ351734–OQ351739 and OQ346357.

**Table 2:** Morphometry of *Oswaldocruzia ukrainae* males.

Characteristics	Togliatti (This Study)	Oktyabrskiy (This Study)	Samara (This Study)	Nikolaevka (This Study)	Lykovshchina (This Study)	Ivanitsky [64]	Svitin [32]	Ryzhikov et al. [3]
*n* ^1^	7	8	3	6	1	1	21	–
Body length	6.20–7.10 (6.71)	6.35–7.83 (7.00)	5.00–5.80 (5.35)	5.20–5.94 (5.58)	5.00	3.94	3.53–5.59 (4.79)	3.8–5.0
Body width	0.110–0.130 (0.119)	0.126–0.160 (0.144)	0.108–0.123 (0.115)	0.111–0.126 (0.118)	0.104	0.140	0.080–0.170 (0.118)	0.088–0.099
Length of cephalic vesicle	0.077–0.085 (0.081)	0.081–0.089 (0.085)	0.079–0.094 (0.087)	0.095–0.111 (0.104)	0.083	0.100	0.075–0.108 (0.091)	0.088–0.099
Width of cephalic vesicle	0.035–0.039 (0.036)	0.038–0.043 (0.040)	0.036–0.040 (0.038)	0.051–0.059 (0.056)	0.041	0.041	0.038–0.048 (0.040)	0.038–0.044
Length of esophagus	0.341–0.377 (0.362)	0.362–0.446 (0.401)	0.346–0.360 (0.353)	0.422–0.454 (0.441)	0.344	0.444	0.358–0.415 (0.380)	0.360–0.390
Width of esophageal bulb	0.045–0.051 (0.048)	0.051–0.059 (0.055)	0.037–0.041 (0.039)	0.039–0.052 (0.045)	0.040	–	0.035–0.055 (0.047)	–
Distance from anterior end of body to nerve ring	0.150–0.173 (0.160)	0.160–0.183 (0.173)	0.165–0.177 (0.172)	0.230–0.250 (0.240)	0.169	–	0.160–0.220 (0.192)	–
Distance from anterior end of body to excretory pore	0.255–0.324 (0.309)	0.320–0.389 (0.349)	0.287–0.310 (0.297)	0.382–0.397 (0.394)	0.330	–	0.250–0.395 (0.346)	–
Tail length	0.102–0.112 (0.106)	0.106–0.118 (0.111)	0.087–0.098 (0.092)	0.092–0.100 (0.097)	0.089	–	0.073–0.113 (0.086)	–
Length of spicules	0.155.167 (0.160)	0.158.170 (0.163)	0.156–0.163 (0.160)	0.148–0.158 (0.154)	0.152, 0.154	0.141–0.160	0.135–0.168 (0.150)	0.143–0.159
Number of crests at mid-body level	33–36	34–37	32–34	33–35	32	22–30	30	–

Here and in Table 3 and Table 4—^1^ number of examined nematodes; measurements in millimeters; mean values are given in parentheses.

**Table 3:** Morphometry of *Oswaldocruzia ukrainae* females.

**Characteristics**	**Togliatti** **(This Study)**	**Oktyabrskiy** **(This Study)**	**Samara** **(This Study)**	**Nikolaevka** **(This Study)**	**Lykovshchina** **(This Study)**	**Svitin [32]**	**Ryzhikov et al. [3]**
*n*	9	11	5	6	2	29	–
Body length	9.95–11.05 (10.47)	10.05–11.55 (10.81)	9.25–10.00 (9.66)	9.25–10.95 (10.11)	9.95–10.48 (10.22)	4.92–12.41 (7.74)	8.00–9.20
Body width	0.161–0.209 (0.183)	0.181–0.217 (0.201)	0.163–0.189 (0.177)	0.184–0.200 (0.192)	0.186–0.198 (0.192)	0.090–0.240 (0.173)	0.130–0.150
Length of cephalic vesicle	0.083–0.102 (0.090)	0.085–0.091 (0.087)	0.083–0.108 (0.092)	0.104–0.113 (0.108)	0.110–0.117 (0.114)	0.070–0.110 (0.091)	0.088–0.100
Width of cephalic vesicle	0.039–0.043 (0.041)	0.041–0.047 (0.044)	0.040–0.044 (0.042)	0.059–0.064 (0.061)	0.056–0.061 (0.059)	0.040–0.053 (0.046)	0.044–0.049
Length of esophagus	0.352–0.413 (0.393)	0.359–0.445 (0.400)	0.350–0.407 (0.374)	0.451–0.476 (0.463)	0.413–0.444 (0.429)	0.355–0.440 (0.395)	0.400–0.450
Width of esophageal bulb	0.051–0.061 (0.056)	0.059–0.067 (0.063)	0.046–0.052 (0.049)	0.045–0.065 (0.053)	0.048–0.52 (0.050)	0.045–0.063 (0.053)	–
Distance from anterior end of body to excretory pore	0.177–0.205 (0.190)	0.158–0.198 (0.179)	0.170–0.200 (0.184)	0.234–0.256 (0.248)	0.227–0.242 (0.235)	0.143–0.238 (0.191)	–
Distance from anterior end of body to vulva	0.320–0.366 (0.348)	0.295–0.369 (0.335)	0.331–0.379 (0.351)	0.420–0.458 (0.444)	0.389–0.422 (0.406)	0.265–0.450 (0.350)	–
Distance from anterior end of body to vulva	6.00–6.75 (6.33)	6.05–7.80 (6.79)	6.17–6.54 (6.38)	6.50–7.10 (6.80)	6.13–6.38 (6.26)	2,87–7.29 (4.61)	–
Tail length	0.172–0.197 (0.185)	0.189–0.217 (0.201)	0.148–0.170 (0.158)	0.165–0.209 (0.191)	0.172–0.180 (0.176)	0.128–0.238 (0.173)	0.140–0.170
Length of eggs	0.081–0.089 (0.085)	0.083–0.091 (0.087)	0.085–0.095 (0.089)	0.085–0.108 (0.096)	0.085–0.098 (0.090)	0.085–0.090	0.099–0.104
Width of eggs	0.045–0.050 (0.047)	0.047–0.051 (0.049)	0.047–0.053 (0.049)	0.050–0.058 (0.054)	0.047–0.055 (0.051)		0.050–0.055
Number of crests at level of mid-body	42–47	43–48	40–44	40–46	42–46	30	–

**Table 4:** Morphometry of *Oswaldocruzia filiformis* from *Bufotes viridis*.

**Characters**	**Spassk** **(This Study)**	**Kirillova et al.** [42]	**Ivanitsky** [64]	**Ryzhikov et al.** [3]	**Kirillova et al.** [42]	**Ryzhikov et al.** [3]
	males				females	
*n*	1	14			2	
Body length	8.65	6.70–9.25 (7.92)	4.00–7.32	5.9–6.2	16.55–17.20 (16.88)	12.0–20.0
Body width	0.163	0.130–0.181 (0.149)	0.125–0.187	0.130–0.160	0.240–0.256 (0.248)	0.230–0.280
Length of cephalic vesicle	0.083	0.075–0.083 (0.079)	0.084	0.077–0.082	0.094–0.098 (0.096)	0.077–0.082
Width of cephalic vesicle	0.041	0.035–0.039 (0.037)	0.050	0.049	0.047–0.051 (0.049)	0.049–0.055
Length of esophagus	0.425	0.378–0.488 (0.431)	–	0.380–0.420	0.559–0.587 (0.573)	0.450–0.490
Width of esophageal bulb	0.051	–	–	–	–	–
Distance from anterior end of body to nerve ring	0.196	–	–	–	–	–
Distance from anterior end of body to excretory pore	0.364	–	–	–	–	–
Distance from anterior end of body to vulva	–	–	–	–	11.06–11.30 (11.18)	–
Tail length	0.139	0.126–0.146 (0.134)	–	–	0.276–0.297 (0.287)	0.260–0.330
Length of spicules	0.228–0.230	0.192–0.236 (0.215)	0.230	0.193–0.321	–	–
Length of eggs	–	–	–	–	0.079–0.087 (0.084)	0.099–0.104
Width of eggs	–	–	–	–	0.039–0.047 (0.044)	0.055
Number of crests at level of mid-body	40	33–40	30–50	–	49–50	–

## Data Availability

GenBank numbers are given in the relevant section of the manuscript. Any other data is available after a reasonable request.
